# Polyfunctional HIV-1 specific response by CD8+ T lymphocytes expressing high levels of CD300a

**DOI:** 10.1038/s41598-020-63025-4

**Published:** 2020-04-08

**Authors:** Joana Vitallé, Iñigo Terrén, Leire Gamboa-Urquijo, Ane Orrantia, Laura Tarancón-Díez, Miguel Genebat, Manuel Leal, Ezequiel Ruiz-Mateos, Francisco Borrego, Olatz Zenarruzabeitia

**Affiliations:** 1 Biocruces Bizkaia Health Research Institute, Immunopathology Group, 48903 Barakaldo, Spain; 2 Clinic Unit of Infectious Diseases, Microbiology and Preventive Medicine, Institute of Biomedicine of Seville (IBiS), Virgen del Rocío University Hospital, University of Seville, CSIC, 41013 Seville, Spain; 3 Laboratory of Molecular Immuno-Biology, Gregorio Marañón University Hospital, Health Research Institute, 28007 Madrid, Spain; 4 Internal Medicine Service, Santa Ángela de la Cruz Viamed Hospital, 41014 Sevilla, Spain; 5 Ikerbasque, Basque Foundation for Science, 48013 Bilbao, Spain

**Keywords:** Cell biology, Immunology

## Abstract

CD300a receptor is found on different CD8+ T cell subsets and its expression has been associated to a more cytotoxic molecular signature. CD300a has an important role in some viral infections and its expression levels are known to be modulated by human immunodeficiency virus (HIV)−1 infection on several cell types. The main objective of this work was to investigate CD300a expression and its regulation during HIV-1 specific CD8+ T cell responses. CD300a receptor expression was analysed by multiparametric flow cytometry on CD8+ T lymphocytes from HIV negative donors, naive HIV-1+ individuals and HIV-1+ subjects under suppressive combined antiretroviral therapy (cART). HIV-1 specific CD8+ T cell response was studied by stimulating cells with HIV-1 derived peptides or with a Gag HIV-1 peptide. Our results showed that HIV-1 specific CD8+ T cells expressing higher levels of CD300a were more polyfunctional showing an increased degranulation and cytokine production. Moreover, we observed an up-regulation of CD300a expression after Gag HIV-1 peptide stimulation. Finally, our results demonstrated an inverse correlation between CD300a expression on CD8+ T lymphocytes and HIV disease progression markers. In conclusion, CD300a expression is associated to a better and more polyfunctional HIV-1 specific CD8+ T cell response.

## Introduction

CD8+ T cells are very important effectors in the control and clearance of viruses through several mechanisms, including granule exocytosis and cytokine production^1–3^. Many reports have shown that CD8+ T cells play a very important role in the control of viral replication during the acute phase of human immunodeficiency virus (HIV)−1 infection, contributing to the initial control of infection^1,3–7^. Thus, a large number of current studies are focused on the search of new therapies with the aim of inducing a potent and effective HIV specific CD8+ T cell response^8–10^.

After antigen recognition and subsequent activation, CD8+ T cells up-regulate the expression of inhibitory receptors with the aim of preventing an excessive response that, if not properly regulated, could be harmful to the host^11,12^. During chronic stimulation, as for example persistent exposure to HIV antigens, CD8+ T cells became progressively dysfunctional and exhausted, and the expression of inhibitory receptors persists^13^. Exhaustion is a process characterized by a loss of proliferative capacity, differentiation and effector functions^12,14,15^. There are several inhibitory receptors that are expressed on exhausted T cells. For instance, programed cell death-1 (PD1) is known to be involved in the regulation of CD8+ T lymphocytes function during chronic HIV-1 infection and their expression correlates with disease progression^11,12,15^. In addition to the negative regulatory role of these inhibitory receptors, they also mark antigen specific T cells. For example, it has been described that PD1 on CD8+ T cells identifies the repertoire of clonally expanded tumor-reactive lymphocytes and situations of chronic inflammation, which is consistent with the T cell receptor (TCR) stimulation-driven of PD1 on T cells^16,17^.

CD300a has been described as another inhibitory receptor expressed on CD8+ T cells, and has been related to different CD8+ T cell-mediated processes^18–20^. Human CD300a is a transmembrane protein with an IgV-like extracellular domain and an intracellular tail containing three classical and one non-classical immunoreceptor tyrosine-based inhibitory motifs (ITIMs) that provide the receptor with an inhibitory capacity^18,21,22^. CD300a is found on the surface of both lymphoid and myeloid cells^18,19,21^. Regarding CD8+ T cells, CD300a is differentially expressed on different subpopulations^18,19^ and mRNA microarray and flow cytometry analysis of CD8+ T cells from HIV negative women showed an association of CD300a expression to a more cytotoxic molecular signature^20^. The ability of human CD300a molecule to inhibit immune processes has been demonstrated in several cell types, including TCR-mediated signalling on CD4+ T lymphocytes^22,23^, B cell receptor (BCR)-mediated signalling^24^, FcεRI and FcRγIIa-mediated signalling on basophils and mast cells^25,26^ and on neutrophils^27^, respectively, and NK cell-mediated cytotoxicity and cytokine production^28–31^.

CD300a recognizes phosphatidylserine (PS) and phosphatidylethanolamine (PE)^30,32–34^. Several publications have shown that the CD300a receptor, and its ligands PS and PE, are involved in viral mechanisms to infect cells and to escape from the immune system attack, as it has been demonstrated, among others, for Dengue virus (DENV) and Pseudorabies virus (PRV), respectively^29,35,36^. Nevertheless, only a few studies have been published describing the role of CD300a molecule during HIV infection. On monocytes, no differences were found in the expression of this receptor between HIV negative donors and HIV-1+ patients under combined antiretroviral treatment (cART)^37^. In contrast, an altered CD300a expression pattern in the lymphoid lineage has been described. HIV-1+ individuals exhibited a lower expression of CD300a on B cells^24^, while they displayed higher CD300a levels on CD4+ T cells^38^, when compared with HIV negative donors. In both, B cells and CD4+ T lymphocytes, cART did not revert the altered CD300a expression in HIV-1 + patients^24,38^. However, a CD4+ T cell subset co-expressing CD300a, PD1 and CD38 was expanded in naïve HIV-1+ individuals, which was found in very low numbers in HIV negative donors and in patients under cART^38^. Interestingly, a negative correlation between CD300a expression on CD4+ T lymphocytes and markers associated with HIV-1 disease progression was discovered^38^. Lastly, we have recently reported that CD300a is up-regulated on CD56^neg^ NK cells from untreated HIV-1+ subjects and importantly, we demonstrated the capacity of CD300a to inhibit antibody-dependent NK cell degranulation and cytokine production in HIV-1+ individuals^31^.

In this work, we have analysed the expression of CD300a inhibitory receptor on CD8+ T lymphocytes from cART naïve and cART-treated HIV-1+ patients and its correlation with markers of HIV-1 disease progression. In addition, we show that high expression levels of CD300a marks a higher polyfunctional HIV-1 specific response by CD8+ T cells in cART naïve HIV-1+ patients. Furthermore, a fast up-regulation of CD300a receptor on HIV-1 specific CD8+ T cells from cART naïve HIV-1+ patients was observed after the stimulation with a Gag HIV-1 peptide.

## Results

### CD300a expression on CD8+ T lymphocytes from HIV-1+ patients

**Figure 1:**
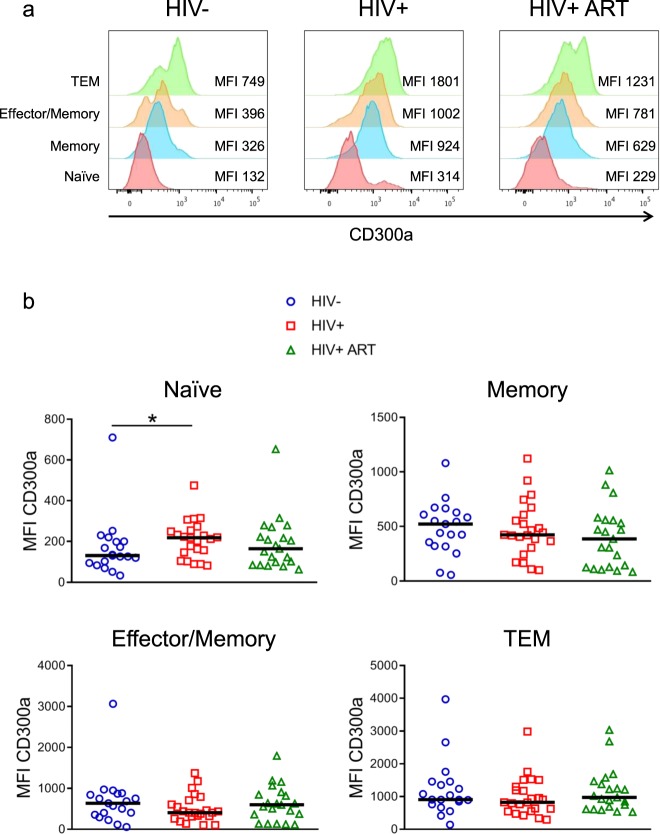
CD300a inhibitory receptor expression on CD8+ T cell subsets from HIV negative donors and HIV-1+ patients. (**a**) Representative histograms showing the median fluorescence intensity (MFI) of CD300a on CD8+ T cell subpopulations. Data from a HIV negative donor, a cART naïve (HIV) and a patient on cART (HIV ART) are shown. (**b**) Dot plots showing the MFI of CD300a on CD8+ T cell subsets from HIV negative donors, cART naïve (HIV) and HIV-1+ patients on cART (HIV ART). Each dot represents a subject and the median is shown. Mann-Whitney test. *p < 0.05.

The CD300a inhibitory receptor has been related to numerous immunological processes and diseases^18,21^ and its expression is known to be modulated on B and CD4+ T lymphocytes from chronically HIV-1+ patients^24,38^. In this work, the first step was to determine the expression of CD300a receptor on CD8+ T cell subsets from HIV-1+ individuals naïve for cART and on cART compared with HIV negative donors. Four CD8+ T cell subpopulations were studied: naïve (CD45RA+ CD27+), memory (CD45RA-CD27+), effector/memory (CD45RA-CD27-) and terminal differentiated effector/memory (TEM) (CD45RA + CD27-) cells (Supplementary Fig. 1a). The frequency of each subpopulation from both HIV negative and HIV-1+ subjects is shown in Supplementary Fig. 1b. To study the expression of CD300a, the median fluorescence intensity (MFI) was taken into account. Regarding to HIV negative people, a differential CD300a expression was observed between CD8+ T cell subsets, as it was described before by our group^19^, and a similar expression pattern was seen in HIV-1+ patients. Specifically, naïve CD8+ T lymphocytes displayed the lowest levels of CD300a expression, memory and effector/memory cells showed intermediate levels and TEM CD8+ T cells exhibited the highest expression (Fig. 1a). When each CD8+ T cell subset from HIV negative donors was compared with the ones from untreated HIV-1+ people and patients under cART, no differences were found concerning CD300a expression levels (Fig. 1b). The exception were naïve CD8+ T cells from untreated HIV-1+ patients, that exhibited higher expression levels of CD300a than naïve CD8+ T cells from HIV negative donors. Effective cART tended to normalize CD300a expression levels on naïve CD8+ T cells (median  HIV- = 131, HIV+ = 215 and HIV+ ART = 157.5) (Fig. 1b).

### Polyfunctional HIV-1 specific response by CD8+ T lymphocytes expressing high levels of CD300a

During acute viral infections, CD8+ T lymphocytes are able to control viral replication by producing cytokines and cytotoxic mediators^3,5^. However, when the infection becomes chronic, a persistent antigen exposure leads to the up-regulation of inhibitory receptors^3,5,12^. Also, at least in certain circumstances, it has been shown that the presence of inhibitory receptors may identify antigen specific chronically stimulated cells^16,17^. Thus, here we investigated the capability of CD8+ T lymphocytes expressing high levels of the CD300a inhibitory receptor to degranulate and produce cytokines in response to HIV-1 peptides.

**Figure 2:**
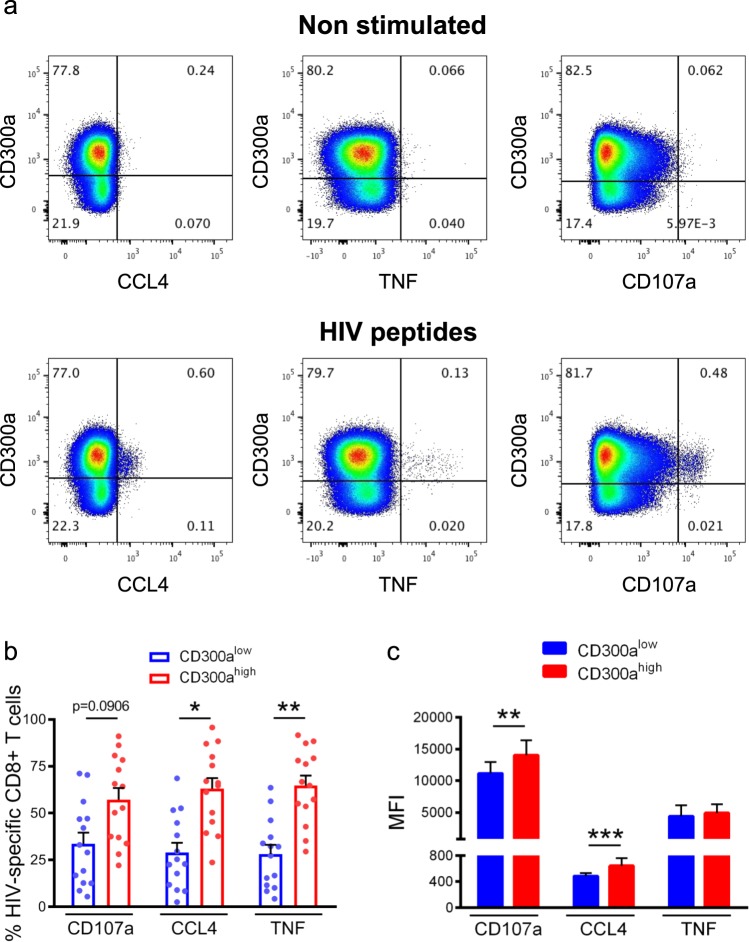
HIV-1 specific CD8+ T cells from cART naïve individuals with higher CD300a expression levels exhibit better degranulation and cytokine production after stimulation. (**a**) Representative pseudocolor dot plots from CD8+ T cells that were non-stimulated (upper panel) or stimulated (lower panel) with a pool of HIV peptides during 6–7 hours. Production of CCL4 and TNF and degranulation (CD107a) were determined. (**b**) Bars and dot plots graph showing the percentage of CD300a^high^ and CD300a^low^ cells within the CD8+ T cells that degranulate (CD107a) and produce CCL4 and TNF in response to HIV-1 peptides. Each dot represents a cART naïve subject. Error bars represent the SEM. (**c**) Bars graph representing the MFI of CD107a, CCL4 and TNF within the CD300a^high^ and CD300a^low^ cells. Wilcoxon matched-pairs signed rank test. Error bars represent the SEM. *p < 0.05, **p < 0.01, ***p < 0.001.

For that, peripheral blood mononuclear cells (PBMCs) from cART naïve individuals were stimulated during 6–7 hours with a pool of HIV-1 derived peptides, followed by the measurement of degranulation (CD107a), and the production of tumor necrosis factor (TNF) and CCL4 (MIP-1β) by CD8+ T lymphocytes from cART naïve HIV-1+ patients. The percentage of positive cells for each parameter was determined based on non-stimulated cells. Our results showed a significant higher frequency of TNF and CCL4 producing cells within CD300a^high^ CD8+ HIV-1 specific T cells in comparison with CD300a^low^ CD8+ T cells. Furthermore, CD300a^high^ CD8+ T cells tended to display also a higher percentage of CD107a+ cells (Fig. 2a,b). When we analysed the MFI of the studied markers within positive cells, similar results were obtained: CD300a^high^ CD8+ T lymphocytes exhibited a higher MFI of CD107a and CCL4 than CD300a^low^ CD8+ T cells (Fig. 2c). These results indicate that CD300a is a marker associated with a higher functional capability of HIV-1 specific CD8+ T cell response in cART naïve individuals.

**Figure 3:**
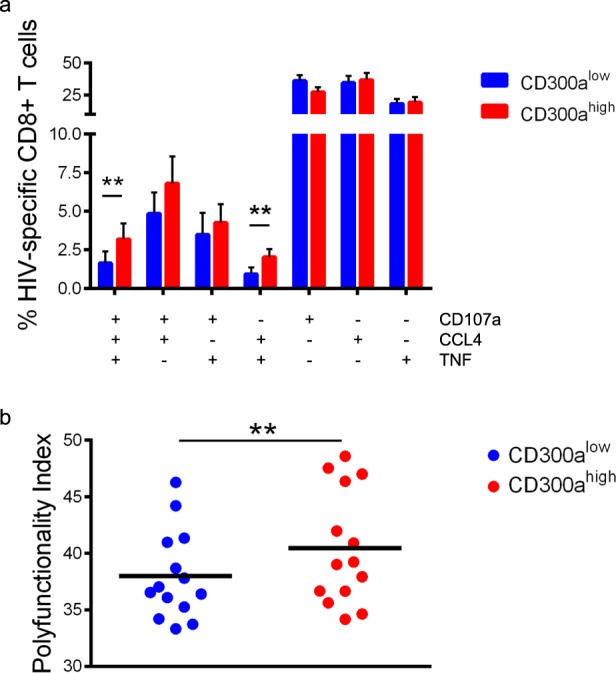
HIV-1 specific CD8+ T cells from cART naïve individuals expressing high levels of CD300a are more polyfunctional. (**a**) Bar graphs showing the percentage of CD300a^high^ and CD300a^low^ cells that are positive for one or more functions (CD107a, CCL4 and TNF) after the stimulation with a pool of HIV-1 peptides during 6–7 hours. Error bars represent the SEM. (**b**) Dot graph showing the polyfunctionality index of CD300a^high^ and CD300a^low^ cells after stimulation with HIV-1 peptides. Each dot represents a cART naïve subject and the median is shown. Wilcoxon matched-pairs signed rank test. **p < 0.01.

The next step was to investigate the polyfunctionality of HIV-1 specific CD8+ T cell response, which is defined as the capacity of cells to produce multiple cytokines and/or degranulate at the same time. In order to study the polyfunctional HIV-1 specific response by CD8+ T lymphocytes expressing CD300a, firstly Boolean gate analysis were carried out. In this situation, only cells that responded to the stimuli were studied. The results showed that after stimulation with HIV-1 peptides, CD300a^high^ CD8+ T cells from cART naïve individuals displayed a higher percentage of cells producing the combination CD107a+ CCL4+ TNF+ and CD107a-CCL4+ TNF+, than CD300a^low^ CD8+ cells (Fig. 3a). As a matter of fact, the same tendency was noticed regarding to CD107a+ CCL4+ TNF- and CD107a+ CCL4-TNF+ cells (Fig. 3a). Moreover, the polyfunctionality index was calculated by using Funky Cells software, which includes a polyfunctionality index algorithm. In line with our previous results, CD300a^high^ CD8+ T lymphocytes showed a higher polyfunctionality index in comparison with CD300a^low^ CD8+ T cells (Fig. 3b). In conclusion, our experiments indicate that the high expression of the CD300a inhibitory receptor marks a subset of CD8+ T lymphocytes more polyfunctional in response to HIV-1 derived peptides in cART naïve patients.

### Up-regulation of CD300a on Gag specific CD8+ T lymphocytes after stimulation

**Figure 4:**
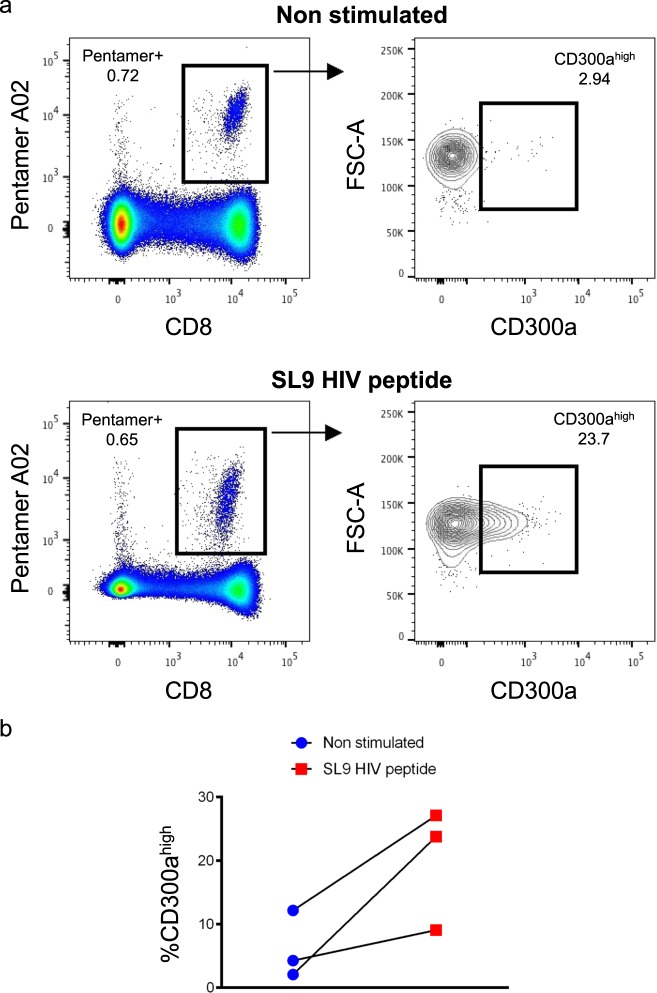
SL9 Gag HIV-1 peptide specific CD8+ T cells from cART naïve people up-regulate CD300a cell surface expression after stimulation. (**a**) Representative example of CD8+ T cells from a cART naïve individual non-stimulated (upper panel) and stimulated (lower panel) with the SL9 Gag (SLYNTVATL) HIV-1 peptide. Specific CD8+ T cells were identified by the staining with the Pentamer A*02:01 loaded with the SL9 HIV-1 peptide (left column). CD300a expression was determined within the Pentamer positive CD8+ T cells (right column). (**b**) Dot graph representing the percentage of CD300a^high^ cells before and after the stimulation with the SL9 HIV-1 peptide. Each dot represents a cART naïve person. Wilcoxon matched-pairs signed rank test.

Once we determined that high CD300a expression on CD8+ T lymphocytes might be associated to a more polyfunctional HIV-1 specific profile, we decided to measure the expression of CD300a on HIV-1 specific CD8+ T cells before and after stimulation with HIV-1 peptides. This will allow us to determine the effect of the specific stimuli in the regulation of the receptor expression. For that, cells from cART naïve HIV-1+ patients were stained with the human leukocyte antigen (HLA)-A*02:01 HIV-1 Pentamer, which allows the detection of SL9 Gag HIV-1 peptide (SLYNTVATL) specific CD8+ T cells. First, we observed within SL9 specific CD8+ T cells low numbers of cells expressing high CD300a expression levels (n = 3, median= 4.29% of CD300a^high^ cells) (Fig. 4). However, we found that after 6–7 hours of stimulation with the SL9 Gag HIV-1 peptide, the percentage of CD300a^high^ cells increased within the SL9 specific CD8+ T cells (n = 3, median= 23.8% of CD300a+ cells). Therefore, our results suggest that CD300a cell surface expression is induced in response to CD8+ T cell activation following recognition of HIV-1 specific peptides.

### Association of CD300a expression with markers of HIV-1 infection progression

**Figure 5:**
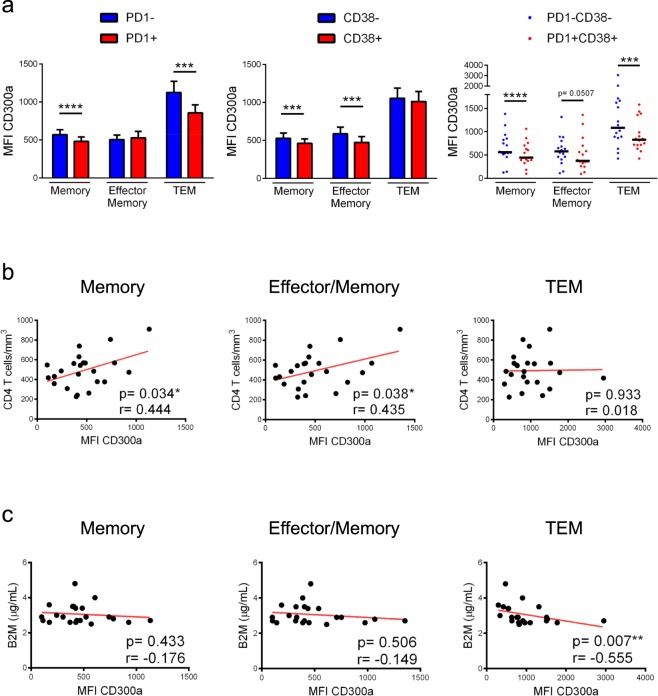
Association of CD300a expression on CD8+ T lymphocytes with markers of HIV-1 infection progression in cART naïve HIV-1+ patients. (**a**) Bars and dot graphs representing the MFI of CD300a within the PD1- and PD1+ cells (left panel), CD38+ and CD38- cells (middle panel) and PD1+ CD38+ and PD1-CD38- cells (right panel) in the indicated CD8+ T cell subsets. Error bars represent the SEM. Each dot represents a donor. Wilcoxon matched-pairs signed rank test. (**b**) Correlation analysis of the MFI of CD300a on memory, effector/memory and TEM CD8+ T lymphocytes, with CD4+ T cell counts. Pearson r test. (**c**) Correlation analysis of the MFI of CD300a on memory, effector/memory and TEM CD8+ T lymphocytes, with β2-microglobulin (B2M) levels. Spearman r test. *p < 0.05, **p < 0.01, ***p < 0.001, ****p < 0.0001.

**Figure 6:**
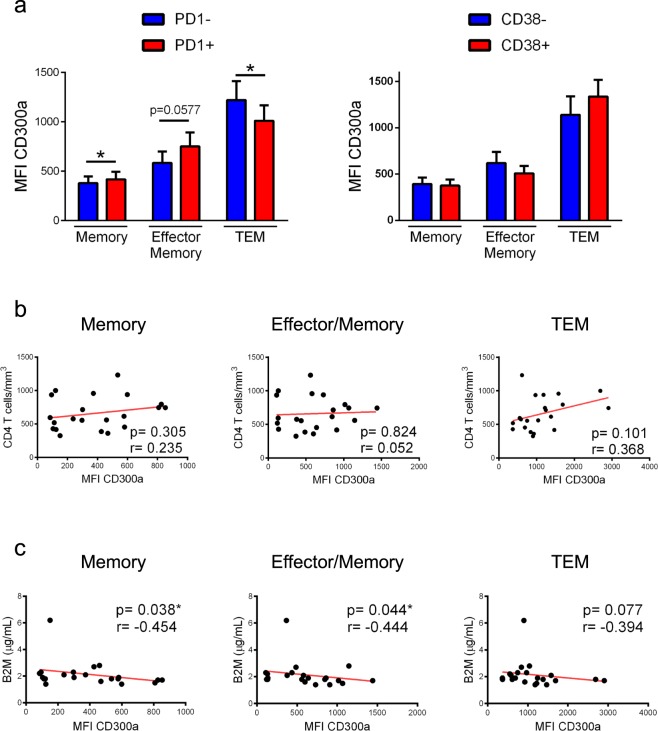
Association of CD300a expression on CD8+ T lymphocytes with markers of HIV-1 infection progression in HIV-1+ patients on cART. (**a**) Bars graphs representing the MFI of CD300a within the PD1- and PD1+ cells (left panel) and CD38+ and CD38- cells (right panel) in the indicated CD8+ T cell subsets. Error bars represent the SEM. Wilcoxon matched-pairs signed rank test. (**b**) Correlation analysis of the MFI of CD300a on memory, effector/memory and TEM CD8+ T lymphocytes, with CD4+ T cell counts. Pearson r test. (**c**) Correlation analysis of the MFI of CD300a on memory, effector/memory and TEM CD8+ T lymphocytes, with β2-microglobulin (B2M) levels. Spearman r test. *p < 0.05.

Next, we decided to investigate if CD300a expression levels on CD8+ T lymphocytes could be associated with HIV-1 disease progression. Several markers of HIV-1 infection progression were analysed. The PD1 inhibitory receptor has been broadly known as a main regulator of T cell exhaustion^39,40^, while the CD38 cell surface receptor is over-expressed on activated T cells^41,42^. The high expression of both, PD1 and CD38, on CD8+ T cells has been reported to be predictors of bad prognosis in HIV-1+ subjects^39,43,44^. Here, we first analysed PD1 and CD38 expression on different CD8+ T cell subsets from HIV-1+ individuals, confirming that our results were in accordance with previous publications (Supplementary Fig. 2a,b)^45,46^. Next, we determined the MFI of CD300a on memory, effector memory and TEM CD8+ T cells expressing PD1 and CD38 in untreated HIV-1+ subjects (Fig. 5) and patients under cART (Fig. 6). Naïve CD8+ T lymphocytes were not included in this analysis, on the one hand, because they express very low levels of PD1 (Supplementary Fig. 2a)^45^, and on the other hand, because they constitutively express CD38 in resting conditions (Supplementary Fig. 2b)^41,46^. Therefore, CD38 is an unsuitable activation marker for naïve CD8+ T cells. Untreated HIV-1+ patients exhibited a lower CD300a expression on PD1+ cells within memory and TEM CD8+ T cells, in comparison with cells that are negative for PD1(Fig. 5a left panel). Similar results were obtained when the CD300a MFI was determined according to the expression of CD38. Memory and effector/memory CD8+ T cells that were positive for CD38 expressed lower levels of CD300a than CD38- CD8+ T cells (Fig. 5a middle panel). Then, we also analysed the MFI of CD300a within CD8+ T cells co-expressing both PD1 and CD38. We observed that PD1+ CD38+ CD8+ T cells expressed significantly lower levels of CD300a than PD1-CD38- CD8+ T cells (Fig. 5a right panel).

Next, correlation analyses were carried out between CD300a expression and clinical markers of HIV-1 infection such as CD4+ T cell count and viral load (Supplementary Table 1). Even though no association was observed regarding viral load (data not shown), a positive correlation was found between CD300a expression levels on memory and effector/memory CD8+ T cells, and CD4+ T cell count in untreated HIV-1+ patients (Fig. 5b). Lastly, different plasma soluble markers from HIV-1+subjects were measured and correlated with CD300a expression, including β2-microglobulin (B2M), high-sensitivity C reactive protein (hsCRP), soluble CD14 (sCD14) and soluble CD163 (sCD163) levels (Supplementary Table 1). Our results did not show any significant association (data not shown), except for a negative correlation that was observed between CD300a expression levels on TEM CD8+ T cells and B2M levels, a marker that reflects immune activation and HIV-1 infection progression (Fig. 5c)^47^.

Afterward, the same analysis was performed on HIV-1+ people on suppressive cART. In general, less significant differences were observed in treated patients when compared with cART naïve individuals. TEM CD8+ T cells expressing PD1 exhibited lower MFI of CD300a in comparison with cells negative for PD1, and effector/memory CD8+ T cells expressing CD38 displayed also lower CD300a expression levels than CD38- CD8+ T cells (Fig. 6a). Focusing on the rest of the CD8+ T cell subsets, no differences were found (Fig. 6a). Since the number of PD1+ CD38+ cells was very low in HIV-1+ patients under cART, this cell population was not studied. Correlation analysis between CD300a expression levels on CD8+ T cells and markers of HIV-1 infection progression described before (Supplementary Table 1) did not show any significant association regarding the treated patients, with the exception of B2M levels that were negatively correlated with CD300a levels on memory and effector/memory subsets (Fig. 6b,c).

Thus, our results showed that CD300a expression levels on CD8+ T lymphocytes are correlated with some disease progression markers, which could suggest an association of high CD300a expression levels on CD8+ T cells with a better prognosis in HIV-1+people. Nevertheless, further experiments are required in order to confirm the predictive value of the expression levels of CD300a receptor.

## Discussion

Upon persistent antigen exposure during chronic HIV-1 infection, CD8+ T lymphocytes undergo a progressive loss of effector functions, which is associated to an up-regulation of several co-inhibitory receptors^3,5,12^. Moreover, it is also known that the expression of these receptors marks antigen specific T cells^16,17^. Undoubtedly, more studies of the numerous surface receptors that modulate CD8+ T cell function are required in order to completely understand the antiviral CD8+ T cell-mediated response. In this work, we have investigated the expression of the CD300a inhibitory receptor on CD8+ T cells belonging to HIV-1+ individuals. Furthermore, we have analysed the HIV-1 specific response by CD8+ T lymphocytes expressing CD300a and the regulation of this receptor expression after the specific stimulation with a Gag HIV-1 peptide in untreated HIV-1+ patients.

CD300a molecule is found on the surface of CD8+ T lymphocytes from both HIV negative adults and newborns, and is expressed at different levels in the four CD8+ T cell subsets^18,19^. Here, we have observed that CD8+ T cells from HIV-1+ subjects exhibited a similar CD300a expression pattern. In fact, no significant differences were seen between cells from HIV negative people and HIV-1+ subjects in most of CD8+ T cell subsets. On the other hand, it has been previously described an altered expression of CD300a in other lymphocytes, including B cells, CD4+ T cells and NK cells, from HIV-1+ individuals when compared with HIV negative people^24,31,38^. Interestingly, other authors have described that CD300a expression on CD8+ T cells is not altered with cytomegalovirus (CMV) infection. Specifically, it was shown that on CD8+ T lymphocytes CD300a increased with age in CMV seropositive individuals, but no differences were found between CMV infected and uninfected people^48^. Instead, in both, HIV-1 and CMV infections, the expression of CD300a is up-regulated on CD4+ T cells^38,48^. These results suggest that CD300a expression on T lymphocytes follows a similar pattern in these two chronic viral infections. Nevertheless, given the high percentage of HIV-1+ patients that are co-infected with CMV^49,50^, further studies are required to determine the role of CMV infection on the regulation of CD300a expression in HIV-1 infection.

HIV-1 infection causes generalized T cell activation, which is reflected in the increased CD38 expression. This activation, instead of resulting in an efficient control of the infection, is predictive of disease progression^41,43,44^. One of the consequences of this chronic T cell activation is the up-regulation of the PD1 inhibitory receptor, which have been associated to immune exhaustion and bad prognosis in chronically HIV-1+ patients^39,40,44^. Quigley *et al*. showed a positive correlation between CD300a mRNA levels and the expression of basic leucine transcription factor ATF-like (BATF) on HIV specific CD8+ T cells^51^. BATF is a transcription factor that is up-regulated in a PD1-mediated signal dependent manner, therefore suggesting that CD300a receptor might contribute to the exhaustion phenotype of CD8+ T cells during HIV infection^51^. In contrast, we have discovered that in cART naïve HIV-1+ people, CD300a expression levels were lower on CD8+ T cells expressing PD1 and cells positive for CD38, in comparison with the PD1- and CD38- CD8+ T cells. Furthermore, CD8+ T cells co-expressing PD1 and CD38 also displayed a lower CD300a expression compared with PD1-CD38- CD8+ T cells from cART naïve HIV-1+ people. In contrast, in most of the CD8+ T cell subsets, no differences were found in the CD300a expression regarding patients under cART. While the expression of PD1 and CD38 are partially reverted with cART, the expression of CD300a is not affected by cART. These results were previously observed in B cells^24^ and CD4+ T cells^38^. An explanation might be that residual effects of HIV infection are sufficient to maintain the levels of CD300a expression, even in the presence of cART. Hence, our results may suggest that CD300a is found at lower levels on dysfunctional CD8+ T lymphocytes in cART naïve HIV-1+ people and therefore, CD300a might not be a marker of exhaustion.

The relationship between HIV-1 infection progression markers and CD300a expression was studied in order to investigate the clinical relevance of this cell surface receptor. No association was found between CD300a expression levels on CD8+ T lymphocytes and viral load, hsCRP, D-dimer, sCD163 and sCD14 plasma levels. However, we did observe a positive correlation with CD4+ T cell count and an inverse correlation with B2M levels. An increase in CD4+ T cell count in HIV-1+ individuals has been broadly considered as good prognosis^52,53^. In contrast, high B2M levels in plasma reveal elevated T cell activation and inflammation, leading to a worse clinical status of the patients^47^. Therefore, it could be conceivable that higher expression levels of CD300a on CD8+ T lymphocytes might be indicative of good prognosis in HIV-1+ patients. Nevertheless, it is clear that prospective studies with larger cohorts will confirm the putative predictive value of CD300a expression on CD8+ T cells from HIV-1+ people.

As commented above, sustained antigen exposure during chronic HIV-1 infection leads to a progressive loss of CD8+ T cell function, consisting of a reduced cytokine production, up-regulation of inhibitory receptors, diminished proliferation and cytotoxicity, defective memory cell generation and, lastly, a deletion of affected cells^12^. Here, we have described a higher capacity of CD300a^high^ CD8+ T lymphocytes to degranulate and produce cytokines in response to HIV-1 derived peptides in cART naïve patients. As a matter of fact, the HIV-1 specific CD8+ T cell response of the CD300a^high^ cells was more polyfuncional. In line with these results, it was previously described that Th1 cells positive for CD300a from HIV negative donors are characterized by a higher polyfunctionality^54^ and that CD300a expression on CD8+ T cells was associated to a more cytotoxic molecular signature^20^. Moreover, these results are also in line with our previous findings showing that CD300a is found at lower expression levels on dysfunctional PD1+ CD38+ CD8+ T lymphocytes from untreated HIV-1+ patients. To conclude, our results suggest that CD300a expression, rather than being a marker of CD8+ T cell exhaustion, is associated to a higher polyfunctional capacity of CD8+ T lymphocytes to respond to HIV-1. This reminds the findings that inhibitory receptors may serve as markers for the identification of clonally expanded lymphocytes in cancer and chronic inflammation^16,17^.

We have also observed an up-regulation of CD300a expression on HIV-1 specific CD8+ T cells after the stimulation with a Gag HIV-1 peptide in cART naïve patients. Given the relative short time (<7 hours) that is required for CD300a to appear on the cell surface after stimulation with the Gag HIV-1 peptide, it would be interesting to determine if this up-regulation requires new protein synthesis or, by the contrary, it may be the result of a translocation from an intracellular pool that goes to the cell surface following activation. Previously, it has been described that stimulation of human neutrophils leads to a translocation of an intracellular pool of CD300a receptors to the cell surface^27^. The reason of CD300a up-regulation after TCR mediated stimulation is not known, but it would not be surprising that this receptor is overexpressed as a regulatory mechanism after CD8+ T cell activation. It is well known that CD300a is able to modulate activation signals on several cell types including mast cells, basophils, neutrophils, eosinophils, NK cells, B cells and CD4+ T cells^18,21^. For instance, co-ligation of TCR and CD300a resulted in a decrease in intracellular Ca^2+^ mobilization in CD4+ T cells, indicating that CD300a inhibits TCR-mediated signals^23^. Importantly, the crosslinking of CD300a with specific monoclonal antibodies (mAbs) is also known to diminish CD16-mediated NK cell degranulation and cytokine production in HIV-1+ individuals^31^. To conclude, we have described an up-regulation of the CD300a inhibitory receptor after the stimulation with a Gag HIV-1 peptide in cART naïve HIV-1+ patients, but undoubtedly more experiments are needed to determine the specific function of CD300a inhibitory receptor on CD8+ T cells effector functions during HIV-1 infection.

In summary, we have studied the expression of CD300a inhibitory receptor on CD8+ T lymphocytes from HIV-1+ patients and its relationship with disease progression markers. Moreover, we have reported a higher and more polyfunctional HIV-1 specific response by CD8+ T cells expressing levels of CD300a and an up-regulation of the receptor expression on HIV-1 specific CD8+ T lymphocytes after the stimulation with a specific HIV-1 peptide. These are encouraging results that hopefully will lead to new research intended to further understand the role of CD300a in CD8+ T cells during HIV-1 infection.

## Methods

### Subjects and samples

For this study, cryopreserved PBMCs and plasma from HIV negative donors and HIV-1+ individuals were used. Samples from 19 HIV negative donors were collected through the Basque Biobank for Research (http://www.biobancovasco.org). The Basque Biobank complies with the quality management, traceability and biosecurity, set out in the Spanish Law 14/2007 of Biomedical Research and in the Royal Decree 1716/2011. The protocol was approved by the Basque Ethics Committee for Clinical Research (PI2014017 and PI2013108). Frozen PBMCs and plasma from asymptomatic HIV-1+ subjects under suppressive cART for at least 6 months (n = 21) (viral load <20 HIV-RNA copies/mL) and from cART naïve HIV-1+ subjects (n = 23) (viral load >9300 HIV-RNA copies/mL) were obtained from Virgen del Rocío University Hospital in Seville (Spain). The study was approved by the Virgen del Rocío University Hospital Ethics Committee for Research (15/2009). All subjects provided written and signed informed consent in accordance with the Declaration of Helsinki, and all methods were carried out in accordance with the relevant ethical guidelines and regulations. Clinical data of HIV-1+ people were also described in a work recently published by our group^38^ (Supplementary Table 1).

### Flow cytometry

For flow cytometry-based procedures, the following fluorochrome-conjugated mouse anti-human mAbs were utilized: PerCP-Cy5.5 anti-PD1 (clone EH12.1), PE-Cy7 anti-CD3 (clone SK7), APC anti-CD38 (clone HIT2), PerCP-Cy5.5 and BV421 anti-CD8 (clone RPA-T8), FITC anti-CCL4 (MIP-1β) (clone D21–1351) and BV421 anti-CD107a (clone H4A3) from BD Biosciences; PE anti-CD300a (clone E59.126) from Beckman Coulter; APC-eFluor780 anti-CD27 (clone O323) from eBiosciences and BV510 anti-CD45RA (clone HI100), PE-Cy7 and APC anti-TNF from Biolegend. To detect dead cells, the LIVE/DEAD Fixable Near-IR Dead Cell Stain Kit (Life Technologies) was used following manufacturer’s protocol.

Frozen PBMCs were thawed at 37 °C and washed twice with phosphate-buffered saline (PBS). Then, the staining of dead cells was done by incubating them with the LIVE/DEAD reagent for 30 min at 4 °C in the dark and they were washed with PBS containing 2.5% of Bovine Serum Albumin (BSA). The staining of CD8+ T cell surface markers was carried out with different fluorochrome-conjugated antibodies for 30 min at 4 °C in the dark. Specifically, CD8+ T lymphocytes were detected by using mAbs against CD3 and CD8, and CD8+ T cell subsets were identified with anti-CD27 and anti-CD45RA mAbs. In addition, mAbs against CD300a, PD1 and CD38 were also added. After the extracellular staining, cells were washed again with 2.5% BSA in PBS and fixed with 200 μl of 4% of paraformaldehide (Sigma-Aldrich) in PBS for 15 min at 4 °C. Lastly, 200 μl of PBS were added and sample acquisition was carried out in a FACS Canto II flow cytometer (BD Biosciences).

For the study of the HIV-1 specific CD8+ T cell function, PBMCs from HIV-1+ individuals were thawed at 37 °C and washed with PBS twice. Then, cells were incubated at 37 °C during 1–2 hours at a concentration of 2 × 10^6^ cells/mL in R10 medium with 10U of DNase (Roche). R10 medium is composed of RPMI 1640 containing GlutaMAX (Thermo Fisher Scientific), 10% Fetal Bovine Serum (Hyclone) and 1% penicyllin/streptomycin (Thermo Fisher Scientific). Afterwards, a wash with R10 was done and cell count was determined. 1–2 × 10^6^ cells in 1 mL of R10 medium were added to FACS tubes and were cultured in the presence or absence of a HIV-1 peptide pool (1 µg/ml per peptide) (ProMix HIV Peptide Pool, ProImmune) or SL9 Gag HIV-1 peptide (10 µg/ml) (SLYNTVATL Custom Peptide, ProImmune) during 6–7 hours. PBMCs that were stimulated with SL9 peptide were previously stained for 10 min at room temperature in the dark, with a HLA class I HIV-1 Pentamer (APC-conjugated HLA-A*02:01 SL9 Pentamer, ProImmune), in order to detect Gag HIV-1 specific CD8+ T cells. After the first hour of incubation, the anti-CD107a mAb and GolgiStop (monensin) and GolgiPlug (brefeldin A) protein transport inhibitors were added according to the manufacturer’s protocol (BD Biosciences). When the stimulation was completed, the staining of death cells and surface markers was carried out as explained above. Then, PBMCs were permeabilized and fixed using Cytofix/Cytoperm Plus Kit (BD Biosciences) following manufacturer’s protocol and were incubated with different fluorochrome-conjugated antibodies during 30 min at 4 °C for the detection of intracellular cytokines. Finally, samples were acquired in the flow cytometer.

### Laboratory methods and ELISA assays

CD4+ T cell counts, viral load and plasma levels of hsCRP, B2M, D-dimer, sCD14 and sCD163 were measured as described in our previous study^38^.

### Data representation and statistical analysis

Data were first analysed with the FlowJo software (version 10.0.7). The graphical representation and statistical analysis were done with GraphPad Prism software (version 6.01). Results were represented in scatter dot plots with the median or bar graphs showing the mean with standard error of the mean (SEM). D’Agostino & Pearson normality test was performed and parametric tests were utilized when data were normally distributed, while non-parametric tests were applied when the normality test was not passed. For the comparison between HIV negative donors, naïve HIV-1+ people for cART and patients under cART, statistical analysis were accomplished by using non-parametric unpaired Mann-Whitney test, and differences between cell subsets from each subject were analysed with non-parametric Wilcoxon matched-pairs test. GraphPad software was also applied to carry out correlation analysis and depending on data distribution, parametric Pearson correlation test or non-parametric Spearman test were employed. To study polyfunctionality of CD8+ T cells, Boolean gate analysis was performed with FlowJo software and these data were represented in bar graphs showing the mean with SEM using GraphPad. Polyfunctionality index was calculated with Funky Cells software^55,56^.

## Supplementary information

Supplementary information.
