# Supplementary material for: Polyfunctional HIV-1 specific response by CD8+ T lymphocytes expressing high levels of CD300a
Source: Sci Rep. 2020 Apr 8;10:6070. doi: 10.1038/s41598-020-63025-4 (PMC7142067; doi:10.1038/s41598-020-63025-4)
Supplement: Supplementary file 1 — Supplementary information. [file 41598_2020_63025_MOESM1_ESM.pdf]

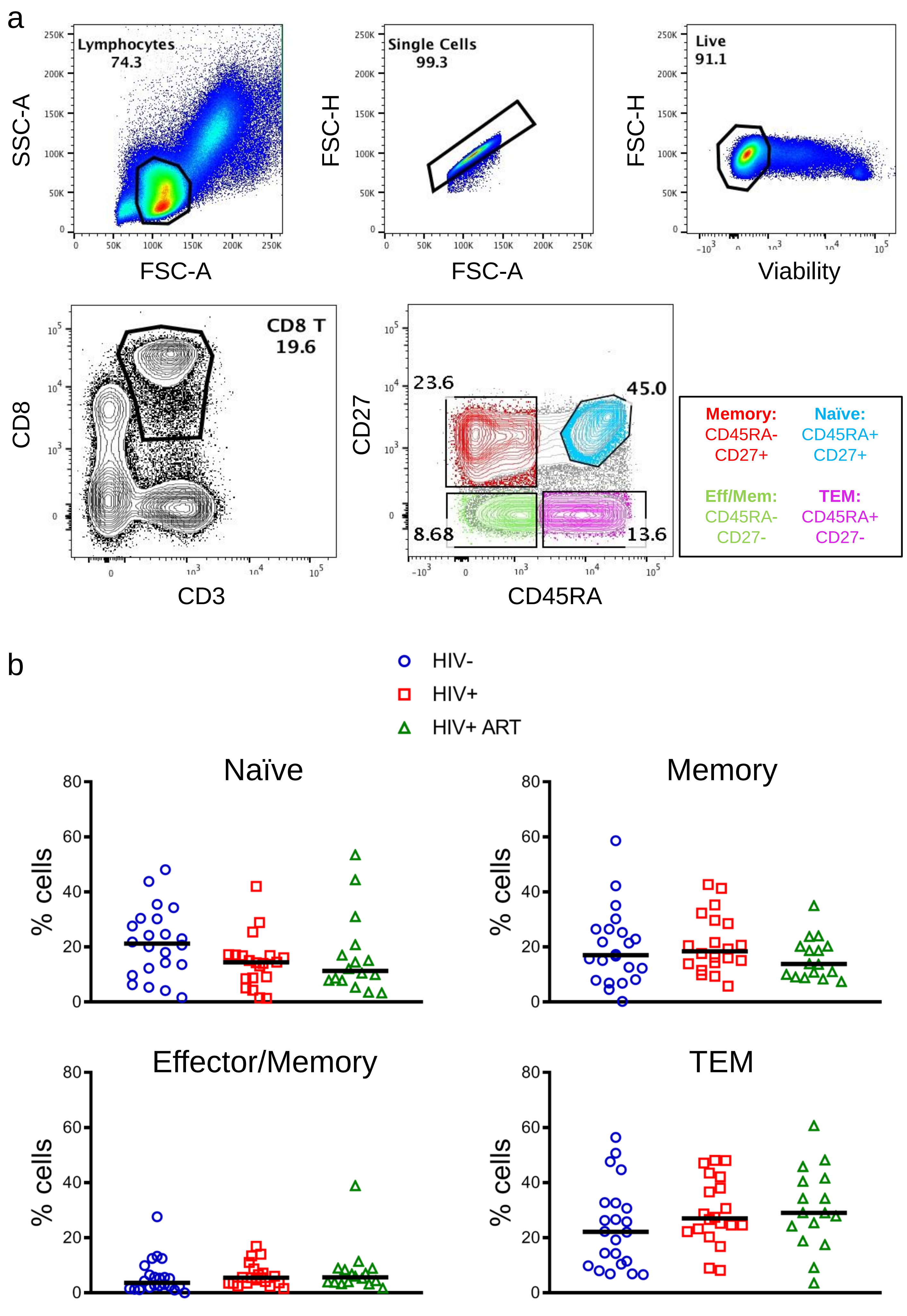

a
Lymphocytes
74.3
SSC-A
FSC-A
Single Cells
99.3
FSC-H
FSC-A
Live
91.1
FSC-H
Viability
CD8 T
19.6
CD8
CD3
23.6
45.0
8.68
13.6
CD27
CD45RA
Memory:
CD45RA-
CD27+
Naïve:
CD45RA+
CD27+
Eff/Mem:
CD45RA-
CD27-
TEM:
CD45RA+
CD27-
b
HIV-
HIV+
HIV+ ART
Naïve
Memory
Effector/Memory
TEM
% cells

Supplementary Fig. 1. CD8+ T cell subsets from HIV-1 negative and HIV-1+ subjects. (a) Pseudocolor and contour plots representing the gating strategy utilized during the study. Data from a representative HIV-1 negative donor is shown. Lymphocytes were electronically gated based on their forward and side scatter parameters, then single and live cells were selected and CD8+ T lymphocytes were detected by the expression of both CD3 and CD8. Four CD8+ T subsets were differentiated based on the expression of CD27 and CD45RA: naïve (CD27+CD45RA+), memory (CD27+CD45RA-), effector/memory (CD27-CD45RA-) and terminal differentiated effector/memory (TEM) (CD27-CD45RA+) cells. (b) Dot plot graphs showing the percentage of each CD8+ T cell subpopulation from HIV-1 negative donors, cART naïve HIV-1+ individuals (HIV) and patients on cART (HIV ART). Each dot represents a subject and the median is shown. Mann-Whitney test.

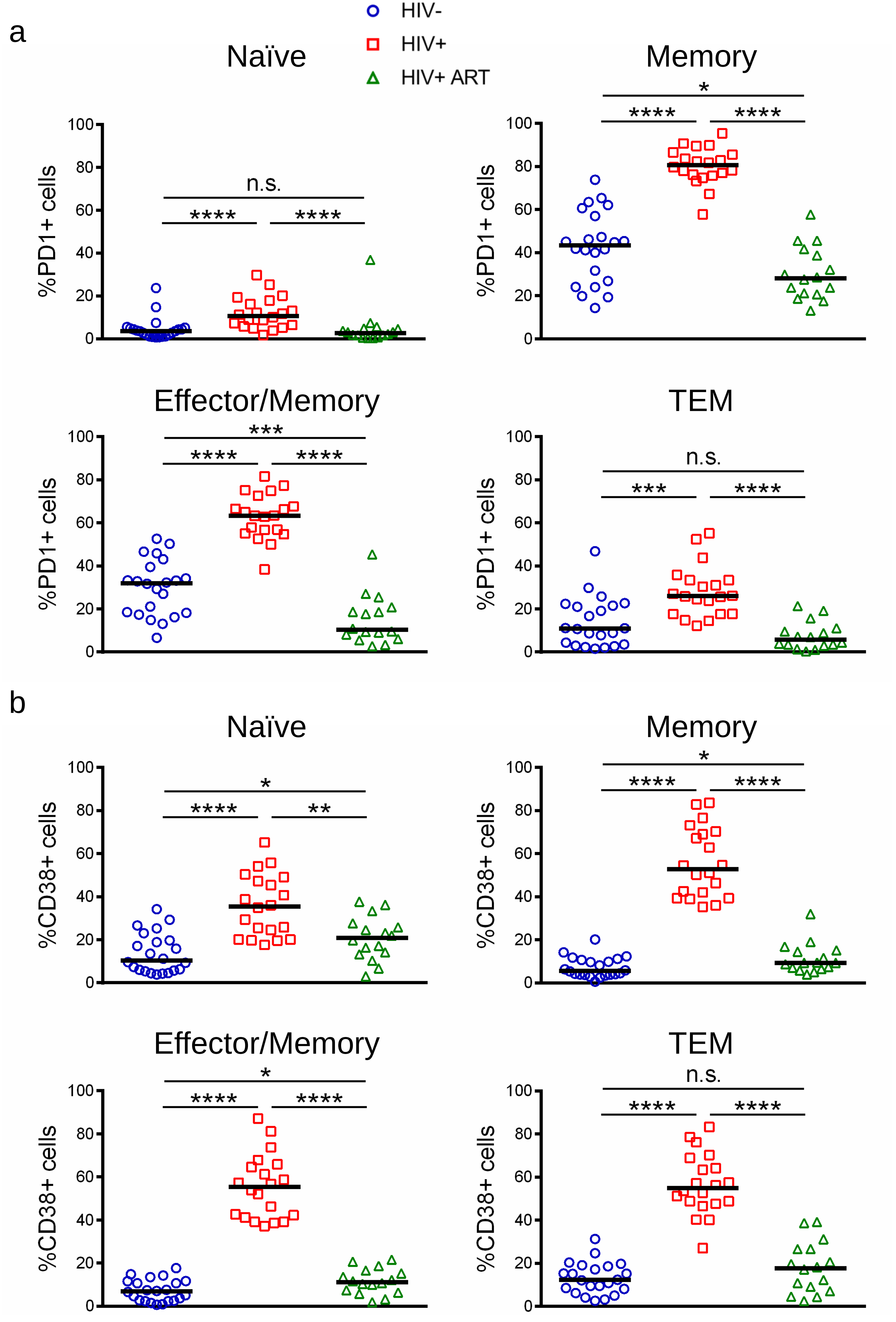

a
HIV-
HIV+
HIV+ ART
Naïve
Memory
Effector/Memory
TEM
%PD1+ cells
n.s.
****
*
***
b
%CD38+ cells
**
0
20
40
60
80
100

Supplementary Fig. 2. (a) Dot plots representing the percentage of PD1+ cells within CD8+ T cells subsets from HIV-1 negative donors, cART naïve HIV-1+ individuals (HIV) and patients on cART (HIV ART). Each dot represents a subject and the median is shown. (b) Dot plots representing the percentage of CD38+ cells within CD8+ T cells from HIV-1 negative donors, cART naïve HIV-1+ individuals (HIV) and patients on cART (HIV ART). Each dot represents a subject and the median is shown. Mann-Whitney test. *$p<0.05$, **$p<0.01$, ***$p<0.001$, ****$p<0.0001$.

Supplementary Table 1. Clinical data and levels of plasma soluble markers from cART naïve HIV-1+ subjects and patients on cART. The median and the range (min-max) are shown. Differences in clinical data and soluble markers between cART naïve HIV-1+ subjects and patients on cART were determined. Mann-Whitney test. *p<0.05, ***p<0.001, ****p<0.0001.

	cART naïve		cART treated		
	**Median**	**Range (min-max)**	**Median**	**Range (min-max)**	**p-value**
**Sex**	Male: n= 22<br>Female: n=1	-	Male: n= 18<br>Female: n=3	-	n.d.
**Age (years)**	29	(20-49)	43	(28-69)	***
**cART (years)**	-	-	4	(2-24)	n.d.
**Viral Load (copies/mL)**	51,900	(6,900-268,000)	<20	-	n.d.
**CD4 T cells/mm^3**	484	(226-910)	596	(325-1,233)	*
**D-dimer (µg/L)**	190	(150-2,320)	150	(150-690)	n.s.
**sCD14 (ng/mL)**	8,445	(5,169-18,134.7)	7,938.8	(5,002.4-16,296.9)	n.s.
**sCD163 (ng/mL)**	1,598.4	(1,075.2-4,217.4)	1,235.3	(823.9-2,554.7)	*
**hsCRP (mg/L)**	2.55	(0.5-20.7)	1.3	(0.4-9.8)	n.s.
**B2M (µg/mL)**	2.9	(2.5-4.8)	1.9	(1.4-6.2)	****

n.d.: not determined; n.s.: not significant
